# The maternal factors during pregnancy for intrauterine growth retardation: An umbrella review

**DOI:** 10.1515/med-2025-1217

**Published:** 2025-06-27

**Authors:** Ensiyeh Jenabi, Hoda Arabzadeh, Sara Abdoli, Salman Khazaei

**Affiliations:** Mother and Child Care Research Center, Hamadan University of Medical Sciences, Hamadan, Iran; Student Research Committee, Hamadan University of Medical Sciences, Hamadan, Iran; Malayer School of Medical Science, Hamadan University of Medical Sciences, Hamadan, Iran; Research Center for Health Sciences, Hamadan University of Medical Sciences, Hamadan, Iran

**Keywords:** umbrella review, risk factor, intrauterine growth retardation, pregnancy

## Abstract

**Background:**

There is no umbrella review on maternal risk factors associated with intrauterine growth retardation (IUGR). Therefore, we assessed an umbrella review on maternal risk factors during pregnancy for IUGR.

**Methods:**

We searched three prominent international databases: PubMed, Scopus, Web of Science, and Cochrane Library. We focused on meta-analyses that examined maternal factors during pregnancy associated with IUGR. The comparison was based on the odds ratio (OR) or related risk ratios reported in the included studies, heterogeneity *I* ^2^, 95% prediction interval, small-study effects, excess significance biases, and sensitive analysis.

**Results:**

Four risk factors for IUGR include placenta previa with an OR of 1.19, placenta abruption with an OR of 2.06, epilepsy in pregnancy with an OR of 1.28, and hepatitis C virus (HCV) infection with an OR of 1.53 were categorized as having suggestive evidence (Class III). Celiac disease with an OR of 2.48 and mullerian anomaly with an OR of 1.93 were considered risk factors with weak evidence (Class IV).

**Conclusion:**

Four risk factors for IUGR including placenta previa, placenta abruption, epilepsy in pregnancy, and HCV infection were categorized as having suggestive evidence (Class III). Celiac disease and Mullerian anomaly were considered risk factors with weak evidence (Class IV).

## 1 Introduction

Intrauterine growth retardation (IUGR), defined as one of the significant complications of pregnancy, is characterized by the fetus having an estimated weight below the tenth percentile [1]. IUGR is observed in approximately 24% of newborns [2]. Almost 30 million newborns suffer from IUGR each year [3]. There are several possible reasons for fetal growth restriction. Sometimes, it is due to issues with the umbilical cord or placenta [4], and the fetus does not receive adequate nutrients and nutrition [5]. Newborns affected by IUGR are at risk of various health problems, diseases, infections, and neonatal mortality [6]. They are also more likely to require prolonged hospitalization [7].

IUGR often has long-term effects on childhood and adulthood [8]. During childhood, an increased risk of cerebral palsy, growth delay, short stature, and neurological disorders can be observed [7]. In adulthood, individuals who have IUGR are more likely to develop high blood pressure, diabetes, obesity, coronary artery disease, stroke, and metabolic syndrome [9]. Common risk factors include maternal causes (hypertension, diabetes, cardiovascular diseases, anemia, malnutrition, smoking, drug use) [10], fetal causes (genetic disorders, congenital anomalies, fetal infections, multiple pregnancies) [11], and placental causes (placental insufficiency, placental infarction, mosaicism) [11]. IUGR is a significant public health concern in developing countries [12], and the identification of risk factors for early interventions is recommended to improve perinatal outcomes and reduce mortality and complications associated with IUGR [4]. In addition, providing ongoing psychological support to women with high-risk pregnancies is emphasized [13].

Some studies have mentioned maternal risk factors during pregnancy that are associated with IUGR. These factors include epilepsy during pregnancy, celiac disease, placenta previa, and placenta abruption [14,15,16,17,18]. By conducting an umbrella review, researchers can obtain a broader understanding of the available evidence, identify agreements or inconsistencies among different systematic reviews, and assess the overall strength and quality of the evidence base on a specific topic. This can be valuable in decision-making, policy development, and identifying areas that require further research. Given that no umbrella review study has been conducted in this field thus far, this study aimed to comprehensively examine meta-analyses of maternal risk factors associated with IUGR.

## 2 Materials and methods

On May 15, 2023, we registered the study protocol for the current umbrella systematic review at the International Prospective Register of Systematic Reviews (PROSPERO). The review was carried out following the Preferred Reporting Items for Systematic Reviews and Meta-Analyses (PRISMA) guidelines [19]. The registration number for the study protocol is CRD42023426661.

### 2.1 Inclusion and exclusion criteria

In the present umbrella review, we included systematic reviews that utilized cohort or case–control designs, as well as cross-sectional. We excluded conference abstracts, letter to the editors, and original articles. Additionally, we excluded genetic factors associated with IUGR and animal studies from our selection process. We focused on meta-analyses that examined maternal factors during pregnancy associated with IUGR. Each meta-analysis was included in our study if it provided the necessary information for a cumulative analysis. Any studies that did not meet this criterion were excluded. If there were two or more meta-analyses related to a factor, we selected the study that included the largest number of original studies.

### 2.2 Search strategy

To retrieve suitable systematic reviews on maternal factors during pregnancy associated with IUGR, we employed a search strategy that involved combining search terms using logical operators (i.e., AND/OR). We searched three prominent international databases: PubMed, Scopus, Web of Science, and Cochrane Library. In addition, Google Scholar was reviewed. The search encompassed articles published from the inception of these databases until May 17, 2023, without any language or time restrictions. The specifics of the search strategy can be found in the supplementary material, specifically in Table S1.

### 2.3 Selection of studies

Two independent authors, E.J. and S.K., conducted the study selection process. They screened the titles and abstracts of the identified studies, as well as the full texts, to determine their suitability for inclusion. Any disagreements that arose during the selection process were resolved through negotiation and discussion.

To ensure comprehensive coverage, the authors also examined the reference lists of potentially relevant studies to identify any additional studies that may have been missed during the initial database search.

For the study selection process, a PECO model (Population, Exposure, Comparison, and Outcome) was utilized. The population of interest consisted of systematic reviews focusing on IUGR in human subjects. The exposure being investigated was the factors associated with IUGR. The comparison was based on the odds ratio (OR) or related risk (RR) ratios reported in the included studies. The primary outcome of interest was exploring the relationship between the identified factors and IUGR.

### 2.4 Extraction of data

Two authors (E.J. and S.K.) independently extracted information from the main body of each article. In case of discrepancies, a third author (H.A.) resolved conflicts. The extracted information included the first author’s name, publication date, sample size, number of included studies in the meta-analysis, effect size, study design type, potential factors, confidence interval, and credibility of the evidence. The *p*-value of the largest study, quality assessment, between-study heterogeneity, and bias were also recorded.

### 2.5 Quality assessment analysis

We employed the “Measurement Tool to Assess Systematic Reviews (AMSTAR2)” to evaluate the quality of the studies included in our analysis [20]. AMSTAR2 resolved disagreements among the assessors. AMSTAR2 comprises 16 questions, as follows:

(1) Was PICO considered in the research question and inclusion criteria?
(2) Was a protocol established beforehand, with any deviations documented?
(3) Was it explained why only specific study designs were included?
(4) Was a comprehensive search conducted?
(5) Was the search performed by two individuals?
(6) Were data extraction and the exclusion list with reasons provided by two individuals?
(7) Were all details of the included papers presented?
(8) Was a proper technique used for assessing the risk of bias?
(9) Were the sources of funding reported?
(10) Were appropriate statistical methods employed?
(11) Was the potential impact of risk of bias in individual studies on the meta-analysis results assessed?
(12) Was the potential impact of risk of bias in individual studies on the discussion of the meta-analysis considered?
(13) Was the heterogeneity of the results of the meta-analysis discussed?
(14) Was an investigation of publication bias conducted?
(15) Were potential influences on the results acknowledged?
(16) Was the presence of potential conflicts of interest reported?

Each of these items will receive a score of “yes,” “partial yes,” or “no.” Notably, items 2, 4, 7, 9, 11, 13, and 15 are rated as critical, carrying higher weight in the scoring process. The cumulative score obtained from these questions will determine the overall quality rating of the meta-analysis, which can be categorized as high, moderate, low, or critically low, offering an overall assessment of the study’s quality and reliability.

### 2.6 Statistical analysis

We conducted all statistical analyses using R software, specifically version 4.0.5. In addition to the R base package, we utilized several other R package, including reporter, Metafor, ConfoundedMeta, xlsx, and epiR. To ensure the accuracy of our analyses, we re-examined the included meta-analyses based on information extracted from the sources, such as reported *p*-values, contingency tables, and sample sizes. In instances where necessary information was not available in the text, we reached out to the corresponding authors of the original papers to request this data. If we did not receive a response, we excluded the original paper from the re-analysis process. For the calculation of pooled effect sizes, we employed a random-effects model during the re-analysis. Throughout our analyses, we adjusted the significance level to 0.05. To assess the heterogeneity among studies, we utilized Cochrane’s *Q* test and the *I* ^2^ measure [21]. Heterogeneity was considered substantial when *I* ^2^ was 50% or higher. Additionally, we computed the prediction interval, which represents the possible range of estimated effect sizes in future studies. Furthermore, we conducted Egger’s regression test to assess the presence of a small study effect [22].

### 2.7 Grading quality of evidence

The classification of the quality of evidence can range from convincing (I) and highly suggestive (II) to suggestive (III), weak (IV), and non-significant (V).

**The criteria for level of evidence classification criteria convincing evidence (Class I):** More than 1,000 cases, significant summary associations (*p* < 10^−6^) per random-effects calculations, no evidence of small-study effects (Egger < 0.1), no evidence of excess of significance bias, prediction intervals not including the null value, largest study nominally significant (*p* < 0.05), not large heterogeneity (*I* ^2^ < 50%), and robust results based on sensitivity analysis.

**Highly suggestive evidence (Class II):** More than 1,000 cases, significant summary associations (*p* < 10^−6^) per random-effects calculation, and largest nominally significant study (*p* < 0.05).

**Suggestive evidence (Class III):** More than 1,000 cases, significant summary associations (*p* < 10^−3^) according to random effect calculations.

**Weak evidence (Class IV):** Random effects *p* < 0.05.

**Non-significant associations (NS):** Random effects *p* > 0.05.

### 2.8 Sensitivity analysis

Mathur’s method was utilized as a sensitivity analysis approach to assess the robustness of the meta-analysis [23]. This method involves calculating a bias factor and the strength of paired confounding associations.

## 3 Results

**Figure 1:**
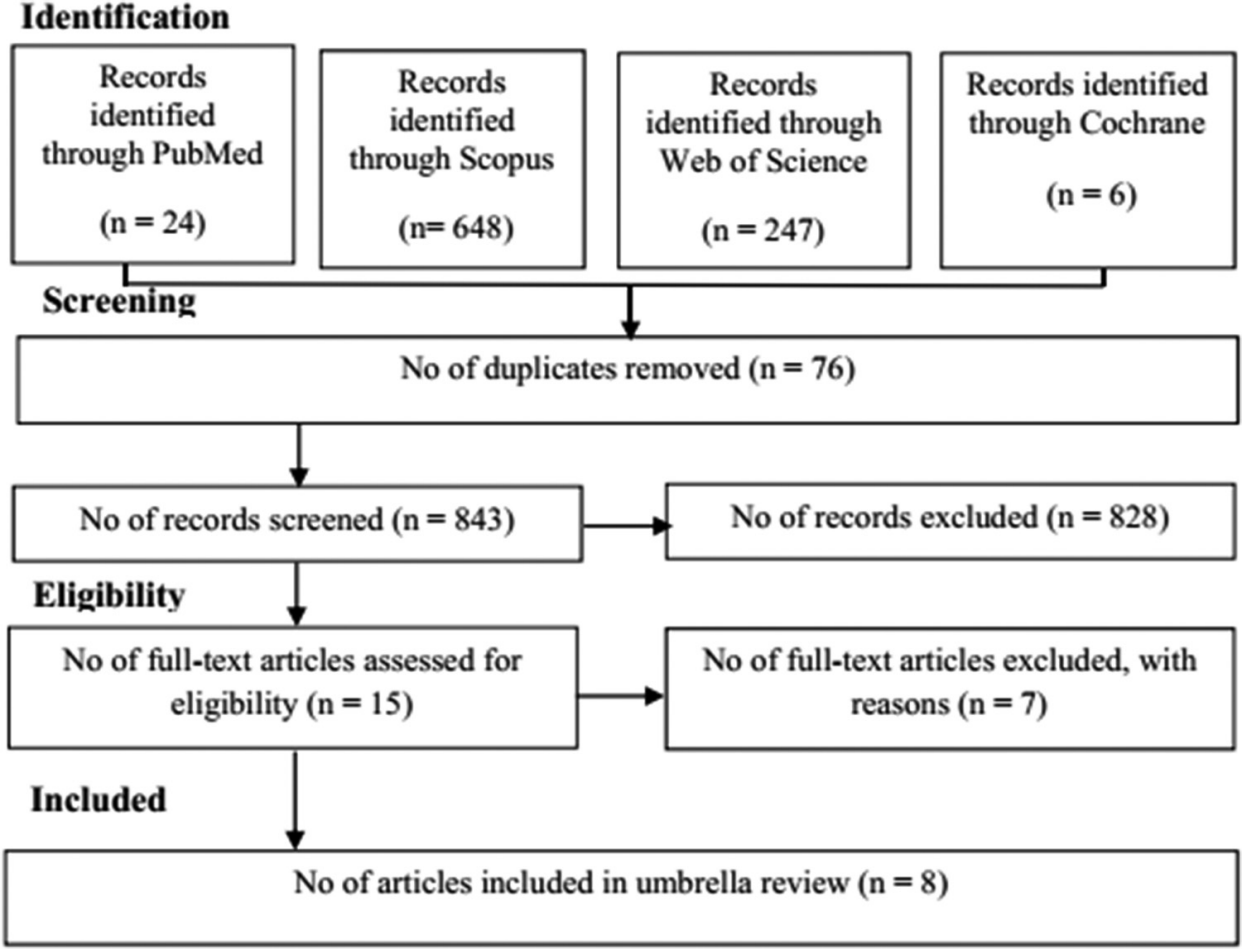
The meta-analyses included in the umbrella review.

In Figure 1, the number of records identified from the PubMed, Scopus, Web of Science, and Cochrane Library databases was 24, 648, 247, and 6 studies, respectively. A total of 82 duplicate records were removed, and after reviewing the titles and abstracts, 828 studies were excluded. Subsequently, 15 studies were selected for full-text evaluation, out of which 8 studies met the inclusion criteria and were included in the final analysis. Reasons for excluding meta-analyses after reading full-text are outlined in Table S2.

In these eight studies, a combined total of 11 meta-analyses were encompassed [12,14,15,16,17,24,25,26], involving 681,159 neonates with IUGR and a staggering 10,433,879 participants.

The studies incorporated in this umbrella review featured cohort, case–control, and cross-sectional designs, comprising 83 original studies, which consisted of 67 cohort studies, 14 case–control studies, and 2 cross-sectional.

In this umbrella review, we identified 11 factors: placenta abruption, placenta previa, celiac disease, mullerian anomaly, subclinical hyperthyroidism, subclinical hypothyroidism (SCH), thyroid peroxidase antibody, isolated hypothyroxinemia, depression during pregnancy, epilepsy in pregnancy, and maternal hepatitis C virus (HCV) infection (Table 1). Four risk factors for IUGR include placenta abruption with an OR of 2.06 (95% CI: 1.57, 2.55), placenta previa with an OR of 1.19 (95% CI: 1.10, 1.27), epilepsy in pregnancy with an OR of 1.28 (95% CI: 1.09, 1.50), and HCV infection with an OR of 1.53 (95% CI: 1.40, 1.68) were categorized as having suggestive evidence (Class III). Celiac disease with an OR of 2.48 (95% CI: 1.32, 4.67) and mullerian anomaly with an OR of 1.93 (95% CI: 1.52, 2.34) were considered risk factors with weak evidence (Class IV). SCH with an OR of 1.54 (95% CI: 1.06, 2.25), thyroid peroxidase antibody with an OR of 1.05 (95% CI: 0.37, 2.92), isolated hypothyroxinemia with an OR of 1.05 (95% CI: 0.37, 2.92), subclinical hyperthyroidism with an OR of 0.98 (95% CI: 0.40, 2.41), and depression during pregnancy with an RR of 1.03 (95% CI: 0.99, 1.08) were not identified as a risk factor for IUGR (Table 1). Among the 11 associations examined in the umbrella review, 6 of them demonstrated statistically significant *p*-values when analyzed using the random-effects model. Importantly, eight studies included at least 1000 IUGR. Additionally, five of these studies reported heterogeneity (*I* ^2^) of less than 50%, one study exhibited small study effects, and four studies displayed an excess significance bias (Table 2).

**Table 1:** The risk factors for intrauterine growth retardation in the umbrella review

Risk factors	Source (year)	Number of population	Number of included studies	Study design	Effect metrics	Random effect summary estimate	AMSTAR1 quality	Credibility of evidence
Placenta abruption	Jenabi (2019)	35,382	7	Case-control, Cohort	Odds ratio	2.06 (1.57, 2.55)	Critically low	Class III
Placenta previa	Balayla (2019)	1,754,836	13	Case-control, Cohort	Odds ratio	1.19 (1.10, 1.27)	Critically low	Class III
Celiac disease	Saccone (2016)	2,823,484	6	Cohort	Odds ratio	2.48 (1.32, 4.67)	Low	Class IV
Mullerian anomaly	Karami (2019)	604,320	7	Case-control, Cohort	Odds ratio	1.93 (1.52, 2.34)	Critically low	Class IV
Subclinical hyperthyroidism	Tong (2016)	6,834	4	Cohort	Odds ratio	0.98 (0.40, 2.41)	Critically low	NS
Subclinical hypothyroidism (SCH)	Tong (2016)	16,257	7	Cohort	Odds ratio	1.54 (1.06, 2.25)	Critically low	NS
Thyroid peroxidase antibody	Tong (2016)	32,874	7	Cohort	Odds ratio	1.05 (0.37, 2.92)	Critically low	NS
Isolated hypothyroxinemia	Tong (2016)	5,013	4	Cohort	Odds ratio	1.05 (0.37, 2.92)	Critically low	NS
Depression during pregnancy	Grote (2010)	1,560	12	Cohort	Relative risk	1.03 (0.99–1.08)	Critically low	NS
Epilepsy in pregnancy	Chen (2017)	962,811	9	Cross- sectional, Cohort	Odds ratio	1.28 (1.09, 1.50)	Critically low	Class III
Maternal hepatitis C virus (HCV) infection	Huang (2016)	4,190,508	7	Case-control, Cohort	Odds ratio	1.53 (1.40, 1.68)	Critically low	Class III

Class III: suggestive; Class IV: weak; NS: not significant.

**Table 2:** The assessment of the evidence credibility for maternal factors association with intrauterine growth retardation

Risk factors	Number of cases	Summary associations (*p*-value) per random-effects calculations	Small-study effects (*p*-value for Egger)	Excess of significance bias (*p*-value)	Prediction intervals	Largest study nominally significant (*p*-value)	Heterogeneity (*I* ^2^%)	Sensitivity analysis	Classification
Placenta abruption	2,357	<0.0001	0.670	0.203	1.03, 8.18	1.7 × 10^−7^	0.0	*T* = 2.83, *G* = 5.12	Class III
Placenta previa	92,412	<0.00001	0.576	0.266	0.44, 4.40	≤0.001	94.0	*T* = 1.27, *G* = 1.86	Class III
Celiac disease	91,552	0.005	0.100	0.996	0.63, 9.81	<0.001	77.0	*T* = 3.63, *G* = 6.73	Class IV
Mullerian anomaly	23,843	0.001	0.904	0.046	0.92, 4.37	<0.001	8.9	*T* = 1.74, *G* = 2.87	Class IV
Subclinical hyperthyroidism	169	0.531	0.630	0.997	0.36, 5.50	0.025	20.8	*T* = 1.56, *G* = 2.49	NS
Subclinical hypothyroidism (SCH)	306	0.072	0.280	0.0008	0.66, 3.88	>0.05	35.1	*T* = 1.84, *G* = 1.65	NS
Thyroid peroxidase antibody	1,976	0.216	0.330	0.034	−0.26, 1.15	0.46	82.9	*T* = 1.45, *G* = 2.26	NS
Isolated hypothyroxinemia	224	0.934	>0.780	0.009	0.18, 6.02	>0.05	60.0	*T* = 1.09, *G* = 1.41	NS
Depression during pregnancy	1,507	0.098	0.008	0.420	0.99, 1.05	0.308	51.0	*T* = 1.18, *G* = 1.64	NS
Epilepsy in pregnancy	111,610	<0.001	0.646	0.500	0.89, 1.90	<0.001	59.9	*T* = 1.26, *G* = 1.83	Class III
Maternal hepatitis C virus (HCV) infection	355,203	<0.00001	0.685	0.256	1.40, 1.68	<0.0001	0.0	*T* = 1.26, *G* = 1.84	Class III

Class III: suggestive; Class IV: weak; NS: not significant.

The sensitivity analyses revealed that four of the meta-analyses (placenta previa, mullerian anomaly, epilepsy in pregnancy, HCV infection) were relatively sensitive to unmeasured confounding, as indicated by a bias factor of less than 1.75 in each of their included studies. These meta-analyses had the potential to decrease the proportion of studies with a true OR exceeding 1.1 to less than 20%. Notably, subclinical hyperthyroidism, SCH, thyroid peroxidase antibody, isolated hypothyroxinemia, and depression during pregnancy did not emerge as a significant risk factor in this context. On the other hand, the remaining two meta-analyses (placenta abruption and celiac disease) demonstrated relative robustness to unmeasured confounding, with a bias factor exceeding 1.90 in each of the included studies. This level of robustness could reduce the percentage of studies with a true OR greater than 1.1 to less than 10% (Table 2).

Importantly, the quality of the included meta-analyses was rated as low critically and a study low based on AMSTAR2 (Table 2 and Table S3).

## 4 Discussion

Based on our current knowledge, this represents the first umbrella review focused on previously published systematic reviews and meta-analyses to evaluate maternal risk factors for IUGR. In this umbrella review, 11 meta-analyses involving 37,425 IUGR and 10,433,879 participants were assessed. The findings from this umbrella review indicate that placenta previa, epilepsy during pregnancy, HCV infection, placental abruption, celiac disease, and Müllerian anomalies are linked to an elevated risk of IUGR.

The evidence level for placenta previa, placenta previa, and HCV infection was classified as suggestive (Class III). This classification was made despite the fulfillment of most criteria, except for the *p*-value, which did not reach the threshold of statistical significance (*p* < 0.000001). It’s worth noting that these classification criteria, though objective and standardized, have faced criticism for their arbitrary cutoffs and susceptibility to misclassification bias [27]. Conversely, it is important to highlight that the evidence derived from meta-analyses is significantly reliant on the methodology and reporting quality of the primary individual studies [28]. The association between celiac disease and IUGR is not entirely clear. Nevertheless, it may be attributed to the malabsorption characteristic of celiac disease, potentially resulting in nutrient deficiencies that are linked to IUGR [29].

In cases of placenta previa, there is a notable disparity in muscle mass and contractile activity between the corpus uteri and fundus, which are significantly greater than those in the lower uterine segment. Consequently, the blood supply to the lower uterine segment is likely reduced, potentially resulting in decreased perfusion for a placenta that is positioned lower in placenta previa. Additionally, recurrent episodes of bleeding from placenta previa can have adverse effects on fetal oxygenation and growth [30]. The cumulative impact of these factors can contribute to IUGR.

The exact mechanisms underlying the impact of placental abruption on IUGR remain unclear. IUGR may result from a sustained response to the chronic damage affecting the placenta. Some evidence has suggested that IUGR is associated with insufficiencies in uteroplacental blood perfusion and ischemia, which may include the presence of placental infarcts [31]. However, it is possible that placental abruption and IUGR can both be attributed to placental dysfunction, potentially stemming from a common cause related to the placenta.

A variety of placental histopathological changes linked to thrombophilia and IUGR were identified, including thrombosis, calcification, infarction, and extensive intravillous or extravillous fibrinoid deposits, occasionally presenting with “canvas-like” features. These changes may arise due to immunogenic factors that disrupt vasculogenesis and compromise placental quality [32]. The association of IUGR as a potential outcome depends on the size of the lesions and the timing of their development during pregnancy [33].

There are several hypotheses suggesting that reduced muscle mass in cases of Mullerian anomalies is linked to IUGR [34]. Consequently, abnormal uterine blood flow and diminished muscle mass may play a role in causing growth restriction associated with Mullerian anomalies [35].

While adult hypothyroid patients tend to gain weight, and hyperthyroid patients typically experience weight loss due to the influence of thyroid hormones on metabolic rates, it is essential to recognize that thyroid hormones in early life play a crucial role in development. Consequently, it is reasonable to consider that thyroid hypofunction, including subclinical cases, could potentially hinder infant development and contribute to IUGR [26].

In cases of epilepsy, it is important to acknowledge that many of the commonly used antiepileptic drugs are transferred to some degree through the placenta [36]. Therefore, when administering treatment, it is crucial to take into account the potential adverse effects (IUGR) of these antiepileptic drugs [37].

Accumulating evidence indicates that imbalances in various vitamins, along with the use of nutraceutical supplements to address these deficiencies, may significantly impact women’s health, particularly in the prevention of IUGR [38]. Maternal malnutrition during pregnancy is also known to hinder embryonic and fetal growth and development, potentially leading to adverse outcomes such as IUGR [39]. Numerous studies have investigated the potential benefits of supplementing with micronutrients such as omega-3 polyunsaturated fatty acids, essential minerals, and vitamins, including folic acid and vitamin D during pregnancy, aiming to reduce the risk of complications like preeclampsia and to prevent fetal growth impairments such as IUGR [40,41].

A study reported that while serum laeverin levels increase during mid-gestation, amniotic fluid laeverin concentrations remain unchanged. Additionally, serum laeverin levels tend to decrease with advancing maternal age, whereas amniotic fluid levels remain unaffected by maternal age. Moreover, laeverin concentrations in both serum and amniotic fluid exhibit mild to moderate correlations with estimated placental volume and fetal weight parameters [42].

Several studies have proposed an association between chronic HCV infection and heightened levels of both local and systemic inflammatory responses [43,44]. In non-diabetic, non-obese populations with HCV infection, a higher ratio of proinflammatory to anti-inflammatory cytokines has been observed in comparison to individuals without HCV infection [45]. Furthermore, it has been shown that excessive inflammation, leading to impaired uteroplacental hemodynamics, plays a pivotal role in the development of IUGR.

### 4.1 Clinical application

Clinicians should take into consideration and discuss these risk factors when counseling their patients. Our umbrella review offers valuable data that can be utilized to provide reassurance to women and guide them toward pre-conception counseling clinics or antenatal clinics.

### 4.2 Strengths and limitations

The primary strength of this study lies in being the first umbrella review on maternal risk factors for IUGR. However, there were several limitations when interpreting the results. To draw a more valid conclusion regarding the association, it is advisable to include additional databases and consider grey literature. Additionally, it is crucial to ensure that the definition and ascertainment of the variables of interest are consistently clear and homogenous across all individual studies. According to the AMSTAR2 checklist, the quality of the included meta-analyses was found to be critically low and, in a study, low. This quality issue could potentially lead to misleading interpretations of the results derived from these meta-analyses. It is worth noting that this review did not encompass other significant risk factors, such as prenatal factors.

## 5 Conclusion

Three to four factors for IUGR including placenta previa, placenta abruption, epilepsy in pregnancy, and HCV infection were categorized as having suggestive evidence (Class III). Celiac disease and Mullerian anomaly were considered risk factors with weak evidence (Class IV). SCH, thyroid peroxidase antibody, isolated hypothyroxinemia, subclinical hyperthyroidism, and depression during pregnancy was not identified as a risk factor for IUGR.

## Supplementary Material

Supplementary Table
