# Supplementary material for: The maternal factors during pregnancy for intrauterine growth retardation: An umbrella review
Source: Open Med (Wars). 2025 Jun 27;20(1):20251217. doi: 10.1515/med-2025-1217 (PMC12205574; doi:10.1515/med-2025-1217)
Supplement: Supplementary Table [file med-2025-1217-sm.pdf]

Supplementary material

Table S1: Search strategy

**Search strategy**
1. Influencing factors or related factors or risk factors
2. *IUGR OR Intrauterine growth retardation OR Fetal growth retardation OR Intrauterine growth restriction*
3. Systematic review or Meta-analysis or Meta analysis or synthesis
4. #1 and #2 and #3

Table S2: Excluded references with reasons

Outcome already explored in larger meta-analysis (*n* = 2)

1. Ghimire U, Papabathini SS, Kawuki J, Obore N, Musa TH. Depression during pregnancy and the risk of low birth weight, preterm birth and intrauterine growth restriction-an updated meta-analysis. Early Hum Dev. 2021;152:105243.
2. Shen GF, Ge CH, Shen W, Liu YH, Huang XY. Association between hepatitis C infection during pregnancy with maternal and neonatal outcomes: A systematic review and meta-analysis. Eur Rev Med Pharmacol Sci. 2023;27(8):3475–88.

IUGR/SGA was reported (*n* = 1)

1. Hill A, Pallitto C, McCleary-Sills J, Garcia-Moreno C. A systematic review and meta-analysis of intimate partner violence during pregnancy and selected birth outcomes. Int J Gynecol Obstet. 2016;133(3):269–76.

IUGR did not estimated with OR/RR (*n* = 1)

1. Littleton HL, Bye K, Buck K, Amacker A. Psychosocial stress during pregnancy and perinatal outcomes: A meta-analytic review. J Psychosom Obstet Gynecol. 2010;31(4):219–28.

There was one study (*n* = 1)

1. Brocklehurst P, French R. The association between maternal HIV infection and perinatal outcome: A systematic review of the literature and meta-analysis. Br J Obstet Gynaecol. 1998;105(8):836–48.

The original articles were not found in any of the databases and search engines (*n* = 2)

1. Li F, Wang T, Chen L, Zhang S, Chen L, Qin J. Adverse pregnancy outcomes among mothers with hypertensive disorders in pregnancy: A meta-analysis of cohort studies. Pregnancy Hypertens.
2. Dapkekar P, Kawthalkar A, Bhalerao A, Somalwar S. Risk factors associated with intrauterine growth restriction: A scoping review. J Datta Meghe Inst Med Sci Univ. 2023;18(1):130–4.

Table S3: Quality of studies based on AMSTAR2 items

	Items																
**Study**	**1**	**2**	**3**	**4**	**5**	**6**	**7**	**8**	**9**	**10**	**11**	**12**	**13**	**14**	**15**	**16**	**Rating**
Balayla (2019)	Y	N	Y	PY	Y	Y	N	PY	Y	Y	Y	N	N	Y	Y	Y	Critically low
Tong (2016)	Y	N	Y	PY	N	N	N	PY	Y	N	Y	N	N	Y	PY	N	Critically low
Saccone (2016)	Y	Y	Y	Y	Y	Y	Y	PY	Y	Y	Y	N	N	Y	Y	Y	Low
Grote (2010)	Y	N	Y	PY	Y	Y	PY	PY	Y	Y	Y	Y	N	Y	Y	Y	Critically low
Chen (2017)	Y	N	PY	PY	N	Y	PY	N	N	N	Y	N	N	N	Y	Y	Critically low
Huang (2016)	Y	N	PY	PY	Y	N	PY	PY	Y	N	Y	N	N	Y	Y	N	Critically low
Karami (2019)	Y	N	Y	PY	N	Y	N	PY	Y	Y	Y	N	N	N	Y	Y	Critically low
Jenabi (2019)	Y	N	Y	PY	N	Y	N	PY	Y	Y	Y	N	N	N	Y	Y	Critically low

Y: Yes; PY: Partial yes; N: No. AMSTAR 2 items evaluated: 1 - PICO consideration in the research question and inclusion criteria. 2 - Protocol establishment beforehand. Any deviations? 3 - Explaining if/why only certain study designs were included. 4 - Comprehensive search. 5 - Two persons performed the search. 6 - Two persons extracted the data. 7 - Providing the list of exclusion with reasons. 8 - Presenting all details of the included papers. 9 - Proper technic for assessing the risk of bias. 10 - Reporting sources of funding. 11 - Appropriate statistical methods. 12 - Assessment of the potential impact of risk of bias in individual studies on the results of the meta-analysis. 13 - Assessment of the potential impact of risk of bias in individual studies on the discussion of the meta-analysis. 14 - Discussion of heterogeneity of the results of meta-analysis. 15 - Investigation of publication bias. Have they influenced the results? 16 - Reports of potential conflict of interest. Items 2, 4, 7, 9, 11, 13, and 15 are the critical items. For High quality: 0–1 non-critical weakness. Moderate quality: >1 non-critical weakness. Low quality: 1 critical flaw with or without non-critical weaknesses. Critically low quality: >1 critical flaw with or without non-critical weaknesses.
